# Foreign Summary

**Published:** 1839-09-14

**Authors:** 


### FOREIGN SUMMARY.

*Cases of Congenital Luxation of the Upper Extremity of the Humerus.* By Robert William Smith, Lecturer on Surgery at the Richmond Hospital School of Medicine.—The following cases, which we extract from a late number of the "Dublin Journal," are examples of a condition of the shoulder-joint, which, so far as we know, has not hitherto been described by authors. Congenital luxation of the head of the humerus presents two varieties, which Mr. Smith distinguishes into the *subcoracoid* and *subacromial*; of the former species he has seen three examples, of the latter but one.

*Congenital subcoracoid luxation.*

*Case* 1.—The first case of original luxation of the head of the humerus was that of Alexander Steele, about twenty years of age; he has been an inmate of the House of Industry, Dublin, for the last four years. He presents an example of congenital displacement of the left shoulder-joint, and upon the same side a specimen of that variety of club-foot termed pes equinus: he does not remember having ever met with any injury of the shoulder; the present condition of the joint has existed from the earliest period of his recollection. The muscles of the shoulder and arm are wasted to a remarkable degree, the circumference of the centre of the arm being three inches and a half less than that of the opposite side; the atrophy has likewise extended to the muscles which pass from the side of the chest to the humerus and scapula, so much so, that the left side of the thorax, measured from the centre of the sternum to a corresponding point posteriorly, is one inch and a half less in circumference than the opposite side of the chest; the trapezius muscle, though not so fully developed as its fellow, still is not wasted to such a degree as the other muscles of the limb; it is the principal muscle which moves the scapula; indeed, it appears to be almost the only one capable of acting upon that bone; the left humerus is nearly half an inch shorter than the right.

The motions of the arm are extremely limited; as it hangs by his side he can merely swing it backwards and forwards, and even in this motion the scapula largely participates; he cannot abduct it in the least, or raise it in any direction; neither can it be abducted by another so far as to bring it to the horizontal line; in the scapula, however, there is a compensatory motion that is very striking; it moves with every motion of the arm; or, perhaps, it would be more correct to say, that the arm follows every motion of the scapula, as the muscles of the former appear to be quite passive, while the trapezius acts strongly upon the latter; indeed, so great is the mobility of the scapula, and so relaxed are its muscles, that when both elbows are pressed upwards simultaneously, and with equal force, the left shoulder can be made to rise between three and four inches above the right. Although the muscles of the forearm are not wasted to such a degree as those of the arm, still great difficulty (owing apparently to the atrophied condition of the biceps) is experienced in flexing the elbow-joint, so as to bring the forearm even to a right angle with the arm; and the means by which the patient does effect it are remarkable: the elevation is not performed gradually, but with a sudden jerk, in which the scapula is also raised considerably, and the arm applied to the side; and sometimes the body is also inclined to the opposite side, and the elbow supported upon the crest of the ileum. The head of the humerus can easily be pressed inwards, so as to allow of the finger being placed in the outer part of the glenoid cavity, and when the bone is pressed outwards towards the acromion, the remainder of the socket can be felt, situated apparently upon a plane posterior to the outer portion; the head of the humerus presents nearly its natural form, as far as can be ascertained by an external examination; the left acromio-clavicular articulation appears to enjoy an unusual degree of motion.

The shoulder has not the rounded form which is natural to it, yet still does not present the flattened appearance which marks the accidental luxation of this joint. The acromion process is prominent, and when the arm hangs by the side, the head of the humerus, distinct and prominent, is so far removed from the under surface of the acromion, that the thumb can easily be placed between them; by raising the elbow this appearance is altogether removed, and the joint assumes more of its natural form, still, however, wanting the rotundity and plumpness derived from a proper development of its muscles.

*Congenital subcoracoid luxation.*

*Case* 2.—Upon the morning of the 3d of April,

1839, I visited Mr. H——, æt. twenty, whose left shoulder-joint presents an example of congenital dislocation under the coracoid process; the appearances are so precisely similar to those detailed in the preceding case, that a full description of them would be useless repetition; it will be sufficient to enumerate a few of the leading characters of the deformity. As the arm hangs by the side, the head of the humerus lies under the coracoid process, and the outer part of the glenoid cavity can be felt beneath the prominent acromion; when the elbow is drawn forwards across the chest, the head of the humerus passes backwards beneath the acromion, vacating completely the abnormal portion of the socket, which can then be plainly felt; the muscles of the shoulder and arm are much wasted, but, as in the case of Steele, the trapezius appears to be as well developed as its fellow of the opposite side; the motions of the arm are very limited; he cannot raise or abduct it, and the motions backwards and forwards are almost the only ones enjoyed; even these are not performed without corresponding movements of the scapula; the deformity has existed since his birth, but became more obvious and striking as he increased in age and stature. For the opportunity of examining this case I am indebted to Mr. Adams.

*Symmetrical subacromial luxations—Congenital.*

*Case* 3.—A woman named Judith Tracy Doyle, æt. forty-two, a lunatic, died on the 8th of last February, in the House of Industry; she had been a patient in the lunatic department of the institution for fifteen years; she was subject to severe epileptic convulsions, in one of which she died. Upon the day following her death I made an examination of the body; the brain presented the appearances so frequently observed in idiots, and so accurately delineated by Cruveilhier; the convolutions of the cerebrum were small and wasted, and the anterior lobes were separated from the frontal bone by an interval of at least three quarters of an inch. When the clothes were removed from the body, I noticed (as did also Mr. Brabazon, who assisted in making the examination) a very singular, and to me, at least, a most unusual appearance of the left shoulder-joint, which, as the body was placed, first caught my eye; the head of the humerus seemed to have been dislocated upon the dorsum of the scapula; but finding that the opposite joint presented a precisely similar appearance, and reflecting upon the very rare occurrence of such an accident, I abandoned this idea, and expressed my opinion at the time, that we had got an example of double *congenital* luxation of the head of the humerus upon the dorsum of the scapula; so perfectly alike were the shoulders, that the description of one will be sufficient.

The coracoid process formed a very remarkable projection, and the acromion was unusually prominent, but still the glenoid cavity could not be felt beneath it; the head of the humerus formed a distinct tumour towards the dorsal surface of the scapula, beneath and behind the summit of the acromion, and closely applied to its inferior surface: the arm was not removed from the side, and the forearm was rotated inwards. Upon examining the interior of the joint, I found that there was no trace of a glenoid cavity in the usual situation, but there was a well-formed socket, surrounded by a glenoid ligament, upon the outer surface of the neck of the scapula; broader above than below, and reaching upwards close to the under surface of the acromion; the tendon of the biceps, perfect throughout, adhered to the upper and inner part of its circumference: the aspect of this abnormal cavity was directed forwards and outwards. The head of the humerus presented the same oval form, with this difference, however, that in the latter case, as already described, the oval form was due to the deficiency of the posterior part of the head, while, in the case of Doyle, it was the anterior portion which was wanting; the lesser tubercle formed a very remarkable projection,—it was elongated and curved, so as to bear considerable resemblance to the coracoid process of the scapula.

*Remarks.*

On the foregoing cases Mr. Smith remarks:—It will be asked, why I consider these cases to present examples of congenital malformation, rather than the consequence of disease or accident? Many circumstances, and much reflection, have induced me to form this opinion. With respect to the first case, that of Steele, it is easy to prove that in his shoulder-joint we have an undoubted specimen of congenital luxation; the boy who is the subject of it never met with any accident, never received any injury of the joint, and it is well known that the condition of the joint which I have described has existed from his infancy, and that the articulation has never been the seat of pain, inflammatory action, or disease of any description: moreover the coexistence of a pes equinus would seem, in some measure, to confirm my opinion as to the nature of the affection of the shoulder.

The symptoms and appearances which have been described by more than one writer, as belonging to an accident termed *partial luxation of the head of the os humeri*, resemble very much those which present themselves in cases of *congenital dislocation* of the same bone, and I feel convinced that examples of the latter have been published under the title of partial luxation; and were it not foreign to the subject of my communication, I could easily show, that the same appellation has been given to a condition of the joint which is obviously the consequence of rheumatic disease.

I should think it scarcely necessary to occupy the time of the reader in attempting to prove that these joints presented examples of congenital luxations of the head of the humerus upon the dorsum of the scapula: the total want of a glenoid cavity in the natural situation, the perfect resemblance between the two abnormal sockets, in form, size, and position; the integrity of the tendons and ligaments, the singular form of the head of the humerus, all confirm this idea. I might also add the very rare occurrence of such an accident as dislocation upon the dorsum of the scapula; few, comparatively speaking, have seen it; and who has witnessed its occurrence in both shoulders of the same person? Sir Astley Cooper mentions that he had met with only two examples of luxation backwards of the humerus in the course of eight and thirty years; and of such rare occurrence did the Baron Boyer think the injury, that to admit of its taking place he supposed there must exist some malformation of the articular surface.

This, as far as I have been able to ascertain, is the only allusion made by any writer to the deficiency of a portion of the glenoid cavity, as a cause of luxation of the head of the humerus.

Whether the dislocation upon the dorsum of the scapula be the result of accident, or the consequence of an original malformation of the glenoid cavity, the external characters of the affection are, as we might expect, similar in both cases; this will be apparent from the following brief examination of the appearances which I observed in the case of Judith Doyle. The transverse diameter of the shoulder, measured from the centre of the clavicle to the head of the humerus, was obviously greater than natural; the exact amount of increase, of course, could not in this case be ascertained, as both shoulders were similarly altered. Still, however, the distance between the two points that I have mentioned struck me at once as being greater than natural; indeed, it is a necessary consequence of the altered position of the head of the humerus. The acromion process did not project so much as it does in the other luxations of the shoulder, neither was the rounded form of the latter as much altered; the flattening was confined altogether to the anterior part of the joint; and what was lost in this direction was gained externally and posteriorly, where a round, firm tumour, indicated plainly the situation of the head of the bone. In the other luxations of the shoulder there is no remarkable projection of the coracoid process; it is, in a measure, obscured by the head of the humerus; but in the case we are considering, nothing could be more striking than the prominence formed by that process, owing, no doubt, to the removal of the head of the humerus from its vicinity. This is a symptom belonging to dislocation upon the dorsum of the scapula, that appears to have escaped the notice of British surgeons, although more than one of the continental writers have enumerated it; it was, I believe, first mentioned by Manne, of Toulon, afterwards by Sedillot, and very lately it has been much dwelt upon by Lepelletier. The arm was directed obliquely downwards and inwards, the elbow approximated to the side, and the hand and forearm in a state of pronation.

Such is a brief but accurate statement of the facts which I have observed relating to congenital luxations of the articulation of the shoulder. Our knowledge of these remarkable affections must, of course, be considered as still incomplete; we want a more extended series of observations; a larger number of cases must be grouped together to enable us to give a full and complete history of congenital dislocations of the shoulder.—*Lancet, abridged from the Dublin Journal.*

---

*Artificial Anus made in the Groin, with success.*—An infant three days old did not present any traces of the anal opening. The raphé of the perineum extended without interruption from the scrotum to the point of the coccyx. The abdomen was tender and tympanitic, but there was no vomiting. The infant had taken the breast several times, and had passed its urine without difficulty. An incision of several lines in length was made over the supposed situation of the anus, and carried to the depth of three quarters of an inch or more, but without success. It was decided then to open the cæcum in the right iliac fossa. An incision was made near the anterior iliac spine; a small knuckle of intestine presented itself, which was replaced, and the cæcum was found without difficulty. It was opened, and several ounces of meconium immediately escaped, followed by a remarkable amelioration of the symptoms. The progress towards cure was very rapid; the alvine evacuations continued to be passed by the artificial opening, and on the eighth day after the operation the sutures were removed.—(*Medizinische Zeitung für Heilkunde in Preusen.*)—*British and Foreign Quarterly.*

---

*Influence of Civilization in the production of Insanity.*—In an elaborate article on this subject, published in the last number of the "Annales d'Hygiene Publique," M. Brierre de Boismont has announced the following propositions:

Insanity is more frequent, and its forms more diversified, in proportion to the degree of civilization to which each nation has attained. It becomes more rare as the nation approaches the savage state.

In civilized nations, insanity commonly depends on moral causes; in uncivilized nations, on physical causes.

At different epochs and in different countries there arise various epidemic forms of insanity, determined by the influence of the dominent ideas of the time.

Any remarkable event, or any great public calamity, is sure to augment the number of deranged persons for the time being.

The increase in the number of insane persons follows closely the development of the intellectual faculties, passions, industry, wealth, and misery.

Insanity being thus shown to depend, in a great measure, on moral causes, and to be closely connected with the march of civilization, it follows that a chief element in its treatment should consist in the application of moral means.

M. de Boismont has illustrated his propositions with a great number of tables, from which we select the following:

| *Capitals.* | *Population.* | *Insane.* | |
|---|---|---|---|
| London . . . . | 1,460,000 | 7,000 | 1.200 |
| Paris . . . . | 890,000 | 4,000 | 1.222 |
| St. Petersburgh . | 377,000 | 120 | 1.3133 |
| Naples . . . . | 364,000 | 479 | 1.759 |
| Cairo . . . . | 330,000 | 14 | 1.30714 |
| Madrid . . . . | 201,000 | 60 | 1.3350 |
| Rome . . . . | 154,000 | 320 | 1.481 |
| Milan . . . . | 150,000 | 618 | 1.202 |
| Turin . . . . | 114,000 | 331 | 1.344 |
| Florence . . . | 80,000 | 236 | 1.338 |
| Dresden . . . | 70,000 | 150 | 1.446 |

*Lancet.*

*Dupuytren's Pommade for the Hair.*—The following formulæ of this pommade are given, each as genuine, in a late number of the "Journal de Pharmacie."

By M. Fontaine.

Beef marrow, four ounces;
Calomel, two drachms and a half;
Alcohol. ext. of cantharides, eighteen grains;
Essence of roses, four drops.

By M. Cap.

Beef marrow, two ounces;
Extract of canthar., eight grains;
Oil of roses, one drachm;
Essence of lemon, four drops.

The following, M. Recluz assures us, was shown to Dupuytren himself, at the Hotel-Dieu, and acknowledged by him to be exact.

Beef marrow, six ounces;
*Nervine balsam, two ounces;
Peruvian balsam, two ounces;
Oil of almonds, an ounce and a half;
Ext. of cantharides, sixteen grains;
Alcohol at 30°, one drachm.

Dissolve the cantharides in the alcohol; melt the marrow and the nervine balsam with the oil, and pass them through a fine filter; then agitate until it acquires the consistence of spermaceti, and add to it the Peruvian balsam, and afterwards the alcoholic solution. When the pommade has well set, fill two pots, containing each two ounces.—*Ib.*

*Albuminous Urine.*—We extract from the last number of the "British and Foreign Quarterly Review," the following table, in which the various causes that may give rise to the presence of albumen in the urine, are pointed out. The writer remarks that,—

"It is, perhaps, impossible to establish any classification of these derangements which shall embrace, under the same head, all those resembling each other in most essential particulars, and separate such as are marked by special characters; but we venture to propose the following, provisionally, as the least objectionable in the existing state of our acquaintance with the subject. A most important distinction between albuminous urines, practically considered, and one which of course exists in nature, is their being thus impregnated during the process of secretion, (which might depend either on a morbid state of the blood, or on a defective exercise of its secretory function on the part of the kidney,) or simply by subsequent admixture; but as it seems hardly possible to decide, in many cases, to which of these categories albuminous urine belongs, we have not adopted it as the basis of our classification. Our fourth class is a full one; some of our readers may probably contend that it should be still fuller.

Albuminuria may be caused by

A. *An abnormal condition of the blood, dependent on*
Scurvy,
Purpura,
Hæmorrhagic eruptive fevers,
Absorption of pus?
———————albuminous or dropsical effusions?

B. *Lesions of genito-urinary apparatus.*
*a.* Functional.
Essential hæmaturia,
Diabetes,
Secretory excitement of urinary organs and passages, produced by } Articles of food. Medicinal agents.
Active renal hyperæmia.
*b.* Organic.
1. Which cause the foreign admixture during the act of secretion:
Acute and chronic simple nephritis,
Pyelitis,
Bright's disease.
2. Which cause impregnation subsequently to the act of secretion:
Blood thrown out in cases of
Contusions, wounds,
Calculous pyelitis,
Cancer of kidney,
Fungous tumours,
Acute cystitis.
Tubercle,
Encephaloid,
Strumous matter,
Pus—*e. g.*, in cases of prostatic abscess,
Muco-pus, in catarrhal inflammation of mucous membrane of urinary passages, especially of the bladder.

C. *Accidental admixture of healthy genito-urinary albuminous products.*
Semen,
Prostatic secretion,
Catamenial fluid.

D. *Doubtful cause.*
Acute febrile affections,
Hysteria?
Scarlatina { Primary fever, Succeeding anasarca,
Gout?
Chronic diseases independent of renal lesion,
Chylous urine.

* For the composition of this balsam, see "Edwards' Manuel," p. 158.—Ed. L.

Such seems to be a tolerably accurate enumeration of the various conditions under which albuminuria has been observed. From the facts related or referred to, the subjoined propositions are immediately derived by the writer.

1. To infer the existence of a special lesion of the kidneys from the mere presence of albuminuria, is utterly incorrect.

2. Consequently, boiling the urine of all the inmates of a hospital, according to the plan of certain observers, in order to determine the frequency of Bright's disease, is liable to lead to false deductions.

3. It will be necessary for future observers to specify the condition of their patients in respect of all the agencies presumed to give rise to a discharge of albumen with the urine, in order to entitle them to refer its appearance indisputably to any one of those agencies rather than another.

4. The characters of the albuminous precipitate itself, as well as those of the urine containing it, must be much more carefully noted than has hitherto habitually been done."

---

*Treatment of Lateral Deviation of the Spine, by division of the Muscles of the Back.* By M. Jules Guerin.—M. Jules Guerin, editor of the "French Medical Gazette," who is, perhaps, the first authority in France on all points connected with deformities of the muscular or osseous system, has recently addressed the following letter to the Academy of Sciences, on a new mode of treating lateral deviations of the spinal column:

I have the honour of acquainting the Academy with the first results of a new operation which I have performed in twelve instances, with success, on patients affected with lateral deviation of the spine. The operation consists in dividing certain muscles of the back and spinal column. Those which I have divided up to the present moment are the trapezius, rhomboideus, levator scapulæ, sacro-lumbalis, longissimus dorsi, and inter-transverse muscles.

I have already demonstrated, in another work, that the majority of the deformities which affect the joints depend on spasmodic muscular retraction, the result of some affection of the nervous centres, or of the nerves distributed to the muscles. This proposition, which has been shown to be generally applicable to deformities of the neck, spine, hip-joint, wrist, and ankle-joint, &c., naturally led to the deduction of two corrollaries,—

1. That the various species of deformity which affect the joints, &c., depend on muscular retraction, affecting variously the different muscles.

2. That the active treatment of each deformity should consist in the division of the muscles or tendons whose retraction gave rise to the specific deformity.

To obtain, however, the object in view, it was necessary to determine with precision the muscles on the retraction of which each deformity might depend; and, on the other hand, show, by actual experiment, that the theory was correct; or, in other words, cure the deformity by the section of the muscles supposed to be affected. This I have done in cases of wry neck and in the different varieties of club-foot, and having extended the practice to lateral deviations of the spinal column, I have demonstrated the truth of the two following propositions:

1. The majority of lateral deviations of the spine depend on active muscular retraction, and their anatomical varieties are but the expression of this retraction occurring in various degrees in the muscles of the spine and back.

2. The active treatment of this order of deformities should consist in dividing (underneath the skin) the several retracted muscles.

The operations which confirm my theory, were performed on individuals of both sexes and of different ages; the youngest being thirteen, the oldest twenty-two years of age. The deviations had all arrived at the second or third degree, with torsion of the spine, and proportionate gibbosity. In some cases, a single section of the retracted muscles was sufficient for the cure; in others I was compelled to operate two or three times. In all cases I obtained immediately after the operation a well-marked degree of straightening of the spinal column; and in one case, that of a young man, twenty-one years of age, who had been treated mechanically for the last eighteen months, the deviation *immediately* disappeared after the division of the longissimus dorsi and corresponding inter-transverse muscles. In all the other cases I was enabled to complete the cure by mechanical means, and my success was constant. In the twelve operations which I have performed no accident of any kind occurred; there was no hæmorrhage; but little pain; no fever; and in all except one union of the wound by the first intention was obtained.—*Lancet, from the French Med. Gaz.*, June 20, 1839.

---

*On Varicocele, and especially on the radical Cure of that Affection.* By H. Landouzy.—Sixty persons out of every hundred are affected with varicocele. Hence the necessity of studying this disease. The term varicocele, as usually employed, includes the two terms varicocele and circocele, the first of which implies an abnormal enlargement of the veins of the scrotum; the last, of those of the spermatic cord, testicle, and epididymis. Varicocele never occurs without circocele, and, in fact, always forms a consequence of it. The term varicocele is employed in preference to that of circocele, and is understood to mean a dilatation of the veins of the scrotum and cord. The age at which it most frequently begins is from ten to thirty. Of forty-five cases, ten of which are reported by others, and thirty-five occurred in the practice of Landouzy himself,

| | | | |
|---|---|---|---|
| 13 were individuals between | 9 and | 15 | years of age, |
| 29 . . . . . | 15 . | 25 | |
| 3 . . . . . | 25 . | 35. | |

The anatomical conditions which dispose to the frequent occurrence of varicocele, are the depending position and great length of the spermatic veins; the weakness of their parietes; the absence of valves; and, especially, the changes in respect to volume, which they are constantly undergoing. We may add to these, the pressure of a column of blood reaching from the second dorsal vertebra to the testicle, and occasional impediments offered by the inguinal canal. The disease is more frequent on the left than on the right side. It is, indeed, extremely rare on the right side, and almost never occurs only on that side. In eight out of seventeen cases, the veins of the right side were enlarged simultaneously with those of the left, but to a much less degree. It is very rarely necessary to operate on the right side. Out of one hundred and twenty operations performed by M. Breschet, one only was on the right side. The chief reasons which have been assigned for the greater frequency of varicocele on the left than on the right side, are the following:—1. The right spermatic veins open into the vena cava in a direction parallel to the axis of that vessel, while the left opens into the left emulgent vein at right angles to the current of blood which flows through it. 2. The greater length of the left spermatic vein. 3. The pressure of the contents of the sigmoid flexure of the colon. With regard to this last cause, Landouzy observes that only one out of seventeen patients was affected by constipation. Amongst the occasional causes of varicocele may be mentioned, all those which either prevent the return of blood to the heart, or determine it in in large quantity to the organs of generation. These need not be particularized. There seems to be no close connexion between varix and varicocele. Of fifteen cases of varicocele, one only was affected with varices, and of twenty persons who had varicose veins in the lower extremities, no single one had varicocele. The symptoms of this disease are slight at first, and its existence is usually discovered by accident. There is a feeling of weight in the testicle, perineum, and loins, and an unusual twitching in the course of the cord; the scrotum is long, pendant, and soft, and increases rapidly in volume under the influence of heat or fatigue. The patient carries the hand, at every instant, to the scrotum, in order to give it a more favourable position. If the patient is not subject to much fatigue, if he does not remain for any length of time in the erect posture, and avoids all the exciting causes of the disease, a suspensory bandage will guarantee him against further suffering. But if the disease is allowed to go on unchecked, it becomes a source of constant suffering. A short walk causes extreme fatigue, the breathing becomes hurried, the face is bathed in sweat, and expresses the deepest distress. In some cases it is impossible to assume the erect posture, without the aid of a suspensory bandage. The case of one of the most celebrated dramatic authors of France is mentioned, who had acquired the habit of composing whilst rapidly pacing his chamber. This disease entirely put a stop to his perambulations, and with them to his literary productions. He was restored by an operation performed by M. Breschet. There is one symptom which our author has never known to be absent, but which has been omitted by other writers on this subject. It is an increased perspiration of the skin of the scrotum on the side affected. This secretion is, in some cases, so abundant as to require the use of a fold of linen. The superficial veins may acquire an enormous size. One case is mentioned in which they equalled, and even surpassed, the volume of the crural vein. Some cases are quoted from Pott and Sir A. Cooper, in which the disease seems to have made a sudden attack; in these instances, Landouzy thinks that the disease had existed in a less marked form, before the acute attack commenced. The atrophy of the testicle, which took place in more than one instance in which varicocele followed an accident, is justly attributable to the accident, and not to the varicocele which was the consequence of it; nevertheless, when the disease is very much advanced, the enlarged veins compress the testicle, and cause the absorption of it. Out of fifteen cases, our author found the testicle in a more or less advanced state of atrophy in nine. The occurrence of atrophy of the testicle, as a consequence of varicocele, is established by quotations from Celsus, Callisen, and Pott. Sir A. Cooper, however, does not seem to have met with any examples. One case, mentioned by Pott, is the only one in which atrophy of both testicles took place, but Landouzy has often observed the right testicle partially atrophied in varicocele of the left side. The atrophy of the testicle is proportioned to the extent of the varicocele. Not so, however, the pain, which is often most considerable where the veins are least enlarged. This fact is attributed to an enlargement of the small veins surrounding some nervous fibres. It is to the acute pain experienced in some cases, and to the constant uneasiness present in all, that the deep melancholy common to almost all diseases of the urinary and genital organs is to be ascribed. The chief object of Landouzy's paper is to prove the superiority of M. Breschet's method of compression to all others which have been recommended. Thirteen cases are related, in all of which great relief, in the majority a perfect cure, was effected by this means. The danger, too, of inflammation of the veins is much less than when other methods are resorted to: the cure, moreover, is accomplished in a less space of time.

As a preliminary step to the performance of M. Breschet's operation for varicocele, it is necessary that the diseased veins should be considerably distended with blood, in order that none of them may escape the compressing action of the forceps; for this purpose, in summer it will be sufficient that the patient should walk for some time previously, but in winter it will be desirable that he should also take a warm bath. This precaution being taken, and the scrotum being previously shaved, the patient stands upright before the surgeon, who, if the varicocele is on the left side, grasps the right side of the scrotum with his left hand, whilst with his right he endeavours to discover the situation of the vas deferens; this is not difficult; its normal situation is at the posterior part of the cord, its form that of a cylindrical stem, equal through its whole extent, its volume that of a large crow-quill, its consistence is hard though elastic, and may be compared to that of a nerve. But the best means of assuring yourself that you hold the vas deferens, is to press it between the fingers, when the patient should feel a peculiar painful sensation, referred both to the testicle and the groin, and which can scarcely deceive either the patient or operator. Having discovered the vas deferens, the operator draws it towards the septum scroti with the thumb and forefinger, and endeavours to separate the veins from it, and to collect them towards the external part of the scrotum. This sort of subcutaneous dissection forms the only difficult part of the operation, and requires patience and attention; the separation of the vessels must be made with the greatest care, in order that no vein should remain with the vas deferens and spermatic artery. The veins being thus separated, an assistant places the first forceps on the upper part of the scrotum, transversely, and as high as possible, but far enough from the root of the penis to prevent the formation of an eschar on it; it will be found convenient to raise the penis against the abdomen. The branches of the forceps should be carried as far as possible towards the septum, excluding the vas deferens, and at the external part of the scrotum a pedicle of skin about two lines in width, and containing capillary vessels only, should be left uncompressed. As soon as the first pair of forceps is properly placed, it should be screwed tight. The second pair should then be placed, in like manner, as low down as possible, without comprising the testicle, and should be tightened in the same way. An improvement in the construction of the forceps is the introduction of a supplementary blade, which may be depressed daily, by means of a screw, so as gradually to increase the pressure without augmenting the pain. It is necessary to be careful, that this increased pressure commences towards the septum, otherwise the vas deferens might be included between the blades. In general, severe pain is felt in the scrotum and groin, immediately after the operation; but this ceases in a few hours, and no further suffering is produced. A compress dipped in cold water should be applied to the scrotum, which should be slightly elevated. The forceps may be removed, from the fifth to the seventh day. The bridle of skin on the outside of the scrotum facilitates much the cicatrization of the wound, the edges of which would otherwise be widely separated by the erections of the penis, and by the weight of the testicle.—*Brit. and For. Med. Rev.*, *from Journal des Connaiss. Méd-Chir.* Jan. Mar. 1838.*

* These papers have since been published as a separate work, by M. Landouzy.

*M. Ricord's Practice in Phimosis.*—He marks out with ink upon the skin of the prepuce, the situation of the dorsum of the corona glandis; a little in front of this mark, he draws two other lines, diverging in the figure of the letter >, reversed, and meeting below the frenum; laying hold of the prepuce with a pair of forceps behind these lines, he, with one sweep of the bistoury, removes the whole; the mucous membrane is then cut as far back as the edges of the retracted incision in the skin. In the next place he divides the frenum of the prepuce, and either ties or cauterises with the nitrate of silver the wounded artery.

M. Ricord is very anxious to impress upon the minds of his audience the necessity of securing the artery of the frenum, either by a ligature or by torsion. If it is attempted to do so by caustic, the surgeon should take care to wipe the end of the vessel quite dry before he applies the caustic, preventing the bleeding by compressing the posterior part with his fingers.—*London Lancet.*

---

*An operation for Cancer in the olden time.*—A cancer in Mrs. Townsend's breast, of Alverston, taken off by two surgeons; one's name was Clerk, of Bridgnorth, another's name was Leach, of Sturbridg. First they cut the skin across and laid itt back, then they workt their hands in ytt, one above and the other below, and so till their hands mett, and so brought itt out. They had their needles and waxt thread ready, but never ust them; and allso their cauterizing irons, but they used them not: she lost not above ℥vi. (six ounces) of blood in all. Dr. Needham coming too late, staid next day to see it opened. Hee said it was a melliceris, and not a perfect cancer; but itt would have been one quickly. There came out a gush of a great quantitie of waterish substance, as much as would fill a flagon; when they had done, they cutt off, one one bitt, another another, and put in a glass of wine and some lint, and so let itt alone till the next day; then they opened it again, and injected myrrhe, aloes, and such things as resisted putrefaction, and so bound itt upp againe.

Every time they dresst itt, they cutt off something of the cancer that was left behind; the chyrurgions were for applying a caustick, but Dr. Needham said no, not till the last, since shee could endure the knife. They prepared her bodie somewhat, and let her blood the day before. One of the chyrurgeons told her afterwards, that shee had endured soe much, that hee would have lost his life ere hee would have suffered the like; and the Dr. said hee had read that women would endure more than men, but did not believe itt till now. The way how and where itt should bee cutt was markt with ink by one Dr. Edwards, who lives at Bridgnorth, Mrs. Townsend likt him very well; hee said iff they could prevent a gangrene, there was little fear, iff shee fell not into a feavour."—*Ward's Diary*, (1648 *to* 1679.)

